# Recent advances in echocardiography: strain and strain rate imaging

**DOI:** 10.12688/f1000research.7228.1

**Published:** 2016-04-29

**Authors:** Oana Mirea, Jurgen Duchenne, Jens-Uwe Voigt

**Affiliations:** 1 Department of Cardiovascular Sciences, KU Leuven – University of Leuven, Herestraat, Leuven, Belgium; 2 Department of Cardiovascular Diseases, UZ Leuven – University Hospitals Leuven, Herestraat, Leuven, Belgium

**Keywords:** echocardiography, strain rate imaging, echocardiographic strain, speckle tracking

## Abstract

Deformation imaging by echocardiography is a well-established research tool which has been gaining interest from clinical cardiologists since the introduction of speckle tracking. Post-processing of echo images to analyze deformation has become readily available at the fingertips of the user. New parameters such as global longitudinal strain have been shown to provide added diagnostic value, and ongoing efforts of the imaging societies and industry aimed at harmonizing methods will improve the technique further. This review focuses on recent advances in the field of echocardiographic strain and strain rate imaging, and provides an overview on its current and potential future clinical applications.

## Introduction

For decades, two-dimensional (2D) and Doppler echocardiography were the central pillars of evaluating left ventricular (LV) function. For purely practical reasons, many clinicians still resort to measurements of LV ejection fraction (EF) as well as the visual analysis of myocardial wall motion when they evaluate LV global and regional performance. However, these methods have a significant inter-observer variability as they depend on the skills and experience of the user. The undisputed need for simple, readily accessible and reliable methods for evaluating function has driven industry towards the development of semi- or fully- automated methods and post-processing tools for quantifying LV function.

Starting from first experiences with tissue Doppler, velocity imaging was followed by deformation imaging. But with the introduction of speckle tracking for analyzing images, quantitative analysis of myocardial function by deformation imaging had its breakthrough into clinical echocardiography. The recent recommendations released by the European Association of Cardiovascular Imaging (EACVI) and the American Society of Echocardiography (ASE) acknowledged the additional value of deformation measurements over traditional functional parameters, such as LV EF, and recommended the technique now for clinical use ^1^. This review will discuss the most recent developments in the field of strain imaging and the application of the method in the clinical setting.

## What is strain?

Strain is defined as the fractional change in length of a myocardial segment relative to its baseline length, and it is expressed as a percentage. Strain rate is the temporal derivative of strain, and it provides information on the speed at which the deformation occurs. Strain is a vector and the complete description of the complex deformation of a piece of myocardium requires three normal and six shear strain components. For practical reasons, the normal strains which are preferred for clinical use are oriented along the coordinate system of the LV; they describe radial thickening and thinning as well as circumferential and longitudinal shortening and lengthening. Lengthening or thickening of the myocardium is represented by positive strain values, whereas negative values represent shortening or thinning. The most commonly used parameter is longitudinal strain, which can be expected to be around 20% in all regions of the LV ^2^.

Strain is ideally suited to quantify myocardial function regionally, but with the introduction of speckle tracking, a new parameter for global LV function assessment called “global strain” has been introduced. In the longitudinal direction, global longitudinal strain reflects the deformation along the entire LV wall which is visible in an apical image. The measurements from all three apical views are combined to give an average GLS value.

It must be noted that myocardial deformation is load-dependent. Therefore, strain and strain rate measurements must be interpreted considering ventricular wall thickness and shape as well as pre- and after-load.

## How to measure strain?

### Tissue Doppler imaging

Tissue Doppler Imaging (TDI) was the first method used for directly measuring myocardial deformation by echocardiography. Since a regional velocity gradient is analytically identical with the temporal derivative of a change in length, strain rate can be directly calculated from two velocity samples at a known distance apart. Integrating strain rate over time results in strain ^3^. The method is well validated ^4^ and has been shown to provide valuable data in a wide range of conditions ^5^. It benefits particularly from the high frame rate of echocardiographic TDI and therefore is the method of choice in all situations where short-lived mechanical events and fast changes in deformation (e.g., in diastole) have to be measured.

### Speckle-tracking echocardiography

Just over a decade ago, speckle-tracking echocardiography (STE) was proposed ^6, 7^ and validated ^8– 10^ as an alternative tool for measuring myocardial function. Observations in large patient populations showed encouraging results regarding its applicability ^8, 11^ in the clinical setting. The tracking algorithm identifies specific myocardial patterns (commonly named “speckles” or “features”) on conventional B-mode echocardiographic images and follows the motion of these patterns frame-by-frame. The potential to track the speckles in any direction within a 2D image allows the calculation of myocardial velocities, displacement, strain and strain rate in any given direction. This multidirectional tracking ability along with its angle independency ^12^ are often regarded as major advantages over TDI. However, STE has also been shown to depend significantly on good image quality and proper image geometry. Because speckle tracking is derived from grey-scale images which have lower frame rates than TDI, measurements of motion and deformation are most reliable for events that last longer, such as systole. Tracking-based measurements of velocity and rate of deformation, however, should only be attempted with caution. The fast and user-friendly (semi-)automated post-processing is the biggest advantage of the technique.

### Three-dimensional speckle-tracking echocardiography

In theory, tracking can be performed in all three dimensions when three-dimensional (3D) echocardiographic image data are available. However, the problems of frame rate and image quality as explained for 2D tracking are potentiated in 3D, while the advantage of a fully three-dimensional deformation analysis remains a hypothesis. Therefore, 3D STE must currently be regarded as an experimental method ^13^.

## Definitions and conventions

To achieve reproducible measurements, the method of measuring strain needs to be defined and communicated together with the result. The definition comprises not only the interrogated deformation component (longitudinal, circumferential, or radial) but also the sampling position within the myocardium (e.g., endocardial, midwall, or full wall) and the temporal definition of the measured parameter, as conventions are lacking in this field and the definitions are dependent on the vendor of the analysis software. In particular, definitions of timing can influence the measurement without being noticed by the user.

Myocardial deformation is a cyclical process, and the definition of when in this cycle the myocardium can be assumed to have reached its “baseline length” is completely arbitrary. However, it is extremely relevant for defining what “zero strain” is. In physiology lessons, we are taught to consider end-diastole as the reference point in the cardiac cycle, but this is a difficult definition as it requires the mitral valve closure to be measured. Therefore, most strain analysis softwares use surrogate parameters such as the R-peak of the QRS complex in the electrocardiogram. The time of the R-peak, however, can deviate considerably from the true mitral valve closure time, in particular when conduction delays are present ^14^.

A similar question occurs when it comes to the definition of end-systole. In physiology, we use the time of aortic valve closure. In strain software, however, several vendors use the nadir of the global strain or volume curve as an approximation, since it can be calculated easily from the tracking data. Again, particularly in conduction delays or regional dysfunction, this surrogate can be very wrong ^14^. Therefore, a good software should allow the measurement of aortic valve closure and its implementation in the strain analysis.

**Figure 1.**
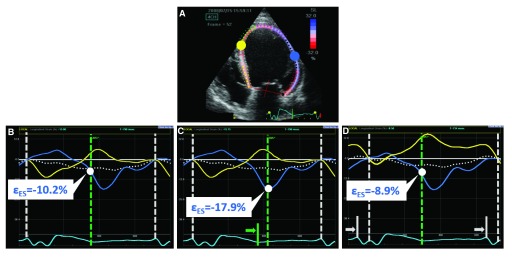
End-systolic strain variability due to changes in timing definitions. **A**) Speckle tracking longitudinal strain in a dilated ventricle with left bundle branch block. The yellow and blue dot indicate the origin of the strain curves of the same colour in the lower panels, where in addition a dotted white curve represents global longitudinal strain. ( **B**) Correct definition of end-diastole (ED) and end-systole (ES). ( **C**) The definition of ES has been moved by + 4 frames (green arrow indicates initial ES; green dashed line indicates current ES). Note the impact on the measured systolic strain value. ( **D**) Here, the definition of ED has been moved by + 4 frames (white arrow indicates initial ED; white dashed line indicates current ED). Also, in this case, the measurement of end-systolic strain is indirectly affected due to a shift in baseline.

**Figure 2.**
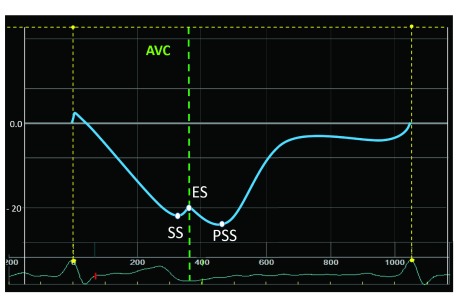
Overview and definition of commonly used strain measurements. Peak systolic strain (SS) is always measured before aortic valve closure (AVC). End-systolic strain (ES) is measured on AVC. Post-systolic strain (PSS) peaks after AVC.

Figure 1 displays the impact of timing changes on strain measurements. Figure 2 shows an overview of commonly used definitions, such as peak systolic strain, end-systolic strain, post-systolic strain (PSS), and peak strain. As can be seen, the definition of end-systole is of particular interest, as derived parameters such as post-systolic shortening directly depend on it.

### Recent clinical applications of deformation imaging

In recent years, functional imaging based on speckle tracking has entered the clinical arena. Hundreds of publications have explored the potential of STE to improve the prognostic and diagnostic accuracy of echocardiography in a wide variety of cardiac pathologies associated with LV dysfunction.

### Prognostic implications

In the general population, lower GLS was shown to be a powerful and independent predictor for vascular events such as stroke and myocardial infarction and for new onset of atrial fibrillation ^15, 16^. GLS also demonstrated prognostic value for adverse outcomes in patients with heart failure ^17– 19^, coronary artery disease (CAD) ^20– 22^, valvular heart disease ^23, 24^, and cardiomyopathies ^25^.

Furthermore, GLS proved to be a superior predictor of all-cause mortality when compared with LV EF and myocardial wall motion in patients with CAD ^26, 27^ or chronic kidney disease ^28^. Longitudinal strain measurements also showed encouraging results in identifying early LV impairment in patients undergoing chemotherapy ^29, 30^ and in subjects with chronic nephropathy ^31^ or diabetes mellitus ^32^.

### Coronary artery disease

Possibly the most valuable clinical application of strain measurements is the evaluation of regional dysfunction in patients with CAD. Early experimental validation against sonomicrometry showed that both Doppler-derived ^4^ and STE-derived ^33^ longitudinal strain can detect the presence of ischemia. Additionally, encouraging data were reported with respect to the ability of strain measurements to predict the extent of the ischemic area ^34^ and to differentiate between transmural and non-transmural scar ^35^.

**Figure 3.**
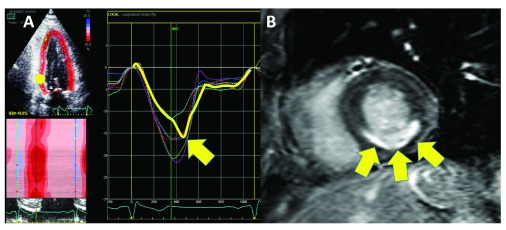
Abnormal deformation in myocardial infarction. ( **A**) Segmental longitudinal strain curves in an apical two-chamber view of a patient with inferior infarction. The bold yellow curve is derived from the infarcted inferobasal segment. Note the pronounced post-systolic shortening (arrow). ( **B**) Cardiac magnetic resonance imaging with delayed enhancement of the same patient. A scar is present in the basal inferior region (arrows). Abbreviations: AVC, aortic valve closure; GS, global strain.

PSS, defined as the presence of regional myocardial shortening after aortic valve closure, is considered a hallmark for myocardial ischemia ( Figure 3). The presence of PSS in areas with ischemic insult has been demonstrated in early studies by using TDI-derived measurements ^36, 37^. A longer persistence of the PSS after an ischemic event was associated with more severe coronary obstruction ^38^. Nevertheless, it is important to consider that although PSS is a very sensitive marker of regional dysfunction, it is never specific for a certain pathology; PSS always needs to be interpreted in a clinical or pathophysiological context or both. PSS at rest can be a sign of ischemia, myocardial scar, or other conditions ^36^. PSS which occurs during stress echocardiography, however, is very likely to be caused by ischemia ^38^, so it can improve the accuracy of detecting CAD during a dobutamine stress test ^39^. Measurements of longitudinal strain during dobutamine stress echocardiography for detecting CAD were reported to be feasible in 75–100% ^40, 41^. Whether these numbers are realistic in a routine clinical scenario, however, remains doubtful.

### LV functional dispersion and dyssynchrony

**Figure 4.**
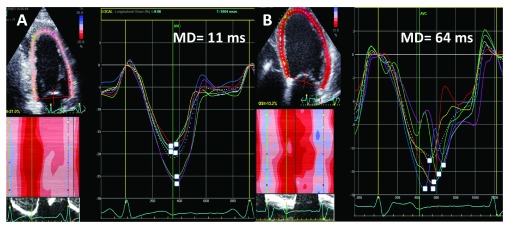
Mechanical dispersion (MD) of the segmental peak longitudinal strain. ( **A**) Normal heart. All strain curves peak around aortic valve closure (AVC). ( **B**) Infarct patient with inducible arrhythmias in the electrophysiology lab. Note the wide dispersion of the segmental peak strains. Abbreviations: GS, global strain.

Strain imaging allows the assessment of asynchronous LV deformation (e.g., by measuring the time to peak strain). Haugaa *et al.* demonstrated in patients with CAD that abnormalities in synchronicity, referred to as mechanical dispersion and defined as the standard deviation of the time to peak regional shortening, could identify those with high risk for arrhythmias ^42^. An example of larger mechanical dispersion due to the presence of ischemia when compared with normal myocardium is presented in Figure 4.

In past years, many indices have been suggested to identify potential responders to cardiac resynchronization therapy (CRT). Initial parameters were based on time-to-peak velocity measurements and could successfully detect dyssynchrony but failed to show added predictive value in prospective clinical trials ^43– 48^. The reason can be found in the inability of velocity measurements to distinguish wall motion due to contraction and wall motion due to tethering which prevents conclusive description of the sequence of wall activation. Later, deformation-based parameters have been suggested, focusing on the timing difference measured between dyssynchronous walls ^49– 54^. Such parameters are potentially useful but only if they identify signatures in the myocardial deformation pattern which are specific for hearts amenable to CRT. More recently, a new index has been proposed which is based on the non-invasive assessment of regional myocardial work and which combines regional LV deformation with an estimate of LV pressure ^55, 56^. It remains to be determined whether such parameters will prove to be superior to the more easy, direct visual or quantitative evaluation of the characteristic motion patterns of dyssynchronous hearts ^57– 59^. Both the fast early systolic inward motion and the subsequent stretching of the septum (septal flash), as well as the rocking motion of the LV apex (apical rocking), can be directly assessed and have been shown to successfully predict CRT response in a clinical setting with both a qualitative and a quantitative approach ^60^.

### Other recent applications

Recent research has demonstrated the complementary character of GLS and EF. Both parameters describe global LV function, but both do it in a different way. Although the parameters follow a linear fit of EF=3|GLS| in most situations ^61^, GLS and EF may diverge depending on the underlying pathology, which could offer added diagnostic information. Several studies have shown that GLS is more sensitive to subtle changes in myocardial function which, for example, could be used in the follow-up of patients receiving chemotherapy ^62, 63^. In hypertrophic pathology, GLS is frequently reduced while EF is still normal ^64^. A higher EF/GLS ratio was found to differentiate cardiac amyloidosis from other pathologies with increased LV wall thickness, such as hypertrophic cardiomyopathy ^65^.

## Advances in the standardization of strain measurements

Establishing the reliability of STE is a prerequisite to its clinical implementation. Although STE has proved to have numerous advantages, such as large availability, high feasibility, and added clinical information, there are still debates regarding the potential discrepancy of strain measurements between vendors ^66, 67^.

In recognition of this problem, the EACVI has initiated a task force together with the ASE and industry partners. The aims of this task force are to identify and minimize sources of variability between strain measurements and to standardize definitions for strain measurements ^3, 68^. The first results of the initiative provide insights on different speckle tracking algorithms, current terminology, and specific technical issues such as image acquisition or timing of measurements ^3^.

In a first comparative study between all major vendors of echocardiography machines and strain software, GLS measurements have been found to show considerable inter-vendor differences, whereas its reproducibility was consistently comparable to or even better than that of conventional echocardiographic parameters ^69^. These findings imply that GLS measurements can be considered a reliable tool in the clinical routine as long as repetitive measurements are performed with the same equipment. On the contrary, the comparison of data obtained with different post-processing software should be avoided.

## Future perspectives

The assessment of LV regional function by speckle tracking has not yet been intensively tested. In particular, vendor-specific differences in the tracking algorithms, such as the number and dimension of kernels, regional smoothing, underlying models of LV motion, or others, may account for more significant differences at a segmental level than at a global level.

Moreover, since regional function can be interpreted through various parameters, the most reproducible and robust for the definition of regional dysfunction remains to be determined. Given the potential value of regional strain measurements, the ongoing efforts of the EACVI/ASE Task Force focus on identifying reasons for the variability of regional strain parameters between vendor-specific software. Positive results would be a remarkable step forward in the process of endorsing regional strain measurements.

## Conclusions

Echocardiographic deformation imaging has developed into an indispensable tool for the clinical assessment of a wide range of cardiac pathologies, and current guidelines recommend its use because of its feasibility and robustness. However, one should remain aware of the pitfalls which still exist and which are easily overlooked because of the user-friendly one-click-gives-it-all approach of most software solutions. Joint efforts of echocardiography associations and industry partners to resolve these pitfalls, to minimize inter-vendor differences, and to standardize measurement definitions will provide a solid valid basis for a widespread use of the technique in the clinical routine.

## Abbreviations

2D, two-dimensional; 3D, three-dimensional; ASE, American Society of Echocardiography; CAD, coronary artery disease; CRT, cardiac resynchronization therapy; EACVI, European Association of Cardiovascular Imaging; EF, ejection fraction; GLS, global longitudinal strain; LV, left ventricular; PSS, post-systolic strain; STE, speckle-tracking echocardiography; TDI, tissue Doppler imaging.
